# Diversity and Homogeneity among Small Plasmids of *Aeromonas salmonicida* subsp. *salmonicida* Linked with Geographical Origin

**DOI:** 10.3389/fmicb.2015.01274

**Published:** 2015-11-23

**Authors:** Sabrina A. Attéré, Antony T. Vincent, Mélanie V. Trudel, Romain Chanut, Steve J. Charette

**Affiliations:** ^1^Département de Biochimie, de Microbiologie et de Bio-informatique, Faculté des Sciences et de Génie, Université LavalQuebec City, QC, Canada; ^2^Institut de Biologie Intégrative et des Systèmes, Université LavalQuebec City, QC, Canada; ^3^Centre de Recherche de l'Institut Universitaire de Cardiologie et de Pneumologie de QuébecQuebec City, QC, Canada

**Keywords:** furunculosis, *Aeromonas salmonicida*, pAsal1, insertion sequence, plasmid stability

## Abstract

Furunculosis, which is caused by *Aeromonas salmonicida* subsp. *salmonicida*, is a major salmonid disease in fish farms worldwide. Several plasmids found in this bacterium confer phenotypes such drug resistance and virulence. Small plasmids (pAsa1, pAsa2, pAsa3, and pAsal1) related to ColE1- and ColE2-type replicons are usually present in its normal plasmidome. In the present study, with the objective to investigate if these plasmids display particularities related to the origin of the isolates bearing them, a total of 153 isolates, including 78 new and 75 previously described, were analyzed for the presence of small plasmids by PCR and DNA restriction fragment profiling. A geographical dichotomy between Canadian and European isolates for their propensity to do not have pAsa3 or pAsal1 was found. In addition, the genotyping analysis led to the identification of two European isolates harboring an unusual pAsal1. An investigation by next-generation sequencing (NGS) of these two isolates shed light on two pAsal1 variants (pAsal1C and pAsal1D). As with pAsal1B, another pAsal1 variant previously described, these two new variants bore a second insertion sequence (IS*AS5*) in addition to the usual IS*AS11*. The characterization of these variants suggested that they could predominate over the wild-type pAsal1 in stressful conditions such as growth at temperatures of 25°C and above. To obtain a comprehensive portrait of the mutational pressure on small plasmids, 26 isolates whose DNA had been sequenced by NGS were investigated. pAsa3 and pAsal1 were more prone to mutations than pAsa1 and pAsa2, especially in the *mobA* gene, which encodes a relaxase and a primase. Lastly, the average copy number of each plasmid per cell was assessed using raw sequencing data. A clear trend with respect to the relative proportion per cell of each plasmid was identified. Our large-scale study revealed a geographical dichotomy in small plasmid repertoire in addition to a clear trend for pAsa3 and pAsal1 to be more frequently altered. Moreover, we present the discovery of two new variants of pAsal1: pAsal1C and pAsal1D.

## Introduction

Fish farming is a major subsistence and economic activity worldwide. However, production-intensive approaches can stress farmed fish populations, increasing their susceptibility to opportunistic pathogens. The waterborne γ-proteobacterium *Aeromonas salmonicida* subsp. *salmonicida* is the causative agent of furunculosis, a major salmonid disease (Dallaire-Dufresne et al., 2014).

Low-resolution approaches such as restriction fragment length polymorphism (RFLP) and random amplified polymorphic DNA (RAPD) analyses initially indicated that *A. salmonicida* subsp. *salmonicida* isolates were homogeneous (Belland and Trust, 1988; Hänninen et al., 1995; García et al., 2000; O'hIci et al., 2000). However, it has recently been shown that most isolates bear variants of a genomic island called *AsaGEI* (Emond-Rheault et al., 2015a,b), suggesting that these variants can be used to determine the geographical provenance of the isolates.

*A. salmonicida* subsp. *salmonicida* not only displays chromosomal diversity, but also variety among its plasmidome. Three small cryptic plasmids (pAsa1, pAsa2, pAsa3) were initially discovered in *A. salmonicida* subsp. *salmonicida* (Belland and Trust, 1989). pAsa1 and pAsa3 are ColE2-type replicons while pAsa2 is a ColE1-type replicon (Boyd et al., 2003). pAsal1, a plasmid putatively derived from pAsa3, was subsequently discovered in *A. salmonicida* subsp. *salmonicida* (Boyd et al., 2003; Fehr et al., 2006). Unlike its homolog pAsa3, pAsal1 is no longer a cryptic plasmid since it bears *aopP*, which encodes a type-three secretion system (TTSS) effector and also bears an insertion sequence (IS) named IS*AS11*. pAsal1B, a recently reported variant of pAsal1 (Trudel et al., 2013) possesses a second IS (IS*AS5*) in addition to the usual IS*AS11*.

As reviewed recently (Piotrowska and Popowska, 2015), *A. salmonicida* isolates may also bear large plasmids involved in bacterial virulence and drug resistance (Adams et al., 1998; L'Abée-Lund and Sørum, 2002; Sørum et al., 2003; Reith et al., 2008; Vincent et al., 2014b), indicating that *A. salmonicida* subsp. *salmonicida* may be an important plasmid reservoir.

In the present study, with the objective to evaluate if variability also exists in the small plasmids repertoire of *A. salmonicida* subsp. *salmonicida* based on the geographical origin of the isolates, we investigated 153 isolates from Canada and Europe by molecular biology screening (PCR and restriction fragment profiling). Twenty-six of which were also analyzed by next-generation sequencing (NGS). Even though most of the isolates harbored the previously described set of small plasmids (pAsa1, pAsa2, pAsa3, and pAsal1), some exhibited differences at the genetic level with a clearly higher tendency of altered profiles for the European isolates. In addition, two of them bore pAsal1 variants and were analyzed further.

## Materials and methods

### Bacterial isolates and growth conditions

We investigated the small plasmid repertoires of 78 new isolates as well as those of 75 previously described (Trudel et al., 2013) to provide a broader portrait of the distribution of small plasmids. The 153 isolates are listed in Table S1. The 01-B526 strain from Canada (Dautremepuits et al., 2006), which contains all the small plasmids (Charette et al., 2012), was used as a control. All the isolates were grown on furunculosis agar for 2 or 3 days at 18°C.

### Plasmid DNA isolation and restriction fragment profiles

Plasmid miniprep kits (Feldan, Canada) were used, as recommended by the manufacturer, to extract < 50-kb plasmids from the isolates listed in Table S1. The extracts (25 μl) were digested with *Eco*RI (New England Biolabs, Canada) (Boyd et al., 2003). The digested DNA samples were separated either by regular gel electrophoresis (0.7%) at 90 V for 75 min or by pulsed field gel electrophoresis (PFGE) with a pulse time ranging from 0.2 to 13.0 s, a run time of 15 h at 6 V/cm, and a 120° angle. The gels were stained with ethidium bromide to visualize the DNA bands under UV illumination.

### PCR analyses

The PCR primers used are listed in Table S2. DNA templates were prepared by lysing one bacterial colony of every *A. salmonicida* isolate in 25 μl of SWL buffer (50 mM KCl, 10 mM Tris, pH 8.3, 2.5 mM MgCl_2_, 0.45% NP-40, and 0.45% Tween 20; Charette and Cosson, 2004). The lysates were heated at 95°C for 5 min. The PCR mixture contained 4 μl of 5X Go-Taq buffer (Promega, USA), 1.6 μl of 2 mM dNTP, 1.3 μl of forward and reverse primers (100 ng/μl of each), 0.1 μl of GoTaq [5 U, (Promega, USA)], 10.7 μl of H_2_O, and 1 μl of DNA template. The PCR program was as follows: 2 min 30 s at 95°C, 30 cycles of 30 s at 95°C, 30 s at 55°C, and 1 min at 68°C, followed by a final 10 min extension at 68°C. For the detection of the IS*AS11* of pAsal1, the PCR program was the same except for the annealing temperature (60°C instead of 55°C). The samples were separated on 1% agarose gels, which were stained with 0.5 μg/ml ethidium bromide. The PCR reactions were performed at least twice.

### DNA extraction and genomic sequencing

The total DNA of 26 selected *A. salmonicida* subsp. *salmonicida* isolates (Table S3) was extracted using DNeasy blood and tissue kits (Qiagen, Canada) and was sequenced by NGS at the Plateforme d'Analyses Génomiques of the Institut de Biologie Intégrative et des Systèmes (IBIS, Université Laval). The DNA libraries were prepared using a KAPA Hyper Prep kit and were sequenced on a MiSeq sequencing system. The sequencing reads were filtered and *de novo* assembled using A5-miseq pipeline version 20140401 (Coil et al., 2014).

In addition, the small plasmids of the JF2506 and JF2507 isolates were extracted using a miniprep kit as indicated above. The purified DNAs were sequenced using a MiSeq apparatus as described above for total DNA. Before performing the *de novo* assembly using A5-miseq version 20150522, the reads were subsampled to obtain an average coverage of approximately 100x. The sequences of pAsal1C (JF2506) and pAsal1D (JF2507) were deposited in the public database GenBank under accession numbers KT781680 and KT781681 respectively.

### Sequence analyses

The sequences of pAsa1, pAsa2, pAsa3, and pAsal1 were assembled and linearized at the same point using tools in EMBOSS version 6.6.0.0 (Rice et al., 2000) in order to compare them. Sequence identity searches were performed with tools in the fasta36 package (Pearson and Lipman, 1988). Annotations of the small plasmid sequences were mainly based on the annotations obtained in a previous study (Boyd et al., 2003) and were performed using Artemis version 16.0.0 (Rutherford et al., 2000). The plasmid sequences were aligned and visualized using Easyfig version 2.1 (Sullivan et al., 2011).

The average copy number per chromosome of each plasmid was determined for each isolate for which the DNA was sequenced. Briefly, the sequencing reads were filtered using Trimmomatic version 0.32 (Bolger et al., 2014) with the parameters suggested in the manual. The resulting filtered reads were mapped on a unique sequence for each plasmid and the house-keeping gene *gyrB* using CUSHAW3 version 3.0.3 (Liu et al., 2014) allowing for 1% mismatches, and the average coverages were computed using Qualimap version 2.1.1-dev (García-Alcalde et al., 2012). The positions of the unique regions were 1..1533 for pAsa1 (GenBank: AY301063.1), 1..1951 for pAsa2 (GenBank AY301064.1), 1..1771 for pAsa3 (AY301065.1), and the *aopP* gene sequence for pAsal1 (GenBank AJ508382.1). The total copy number of each plasmid was determined by comparing the average coverages of the plasmids with the coverage of *gyrB*, for which there was only one copy per chromosome. The statistical analyses were performed using the statistical framework R (R Core Team, 2013).

## Results and discussion

### Small plasmid repertoires

Of the 153 isolates analyzed (see Table S1), 21 displayed variations in their small plasmid repertoires (Table 1). Regrouping the isolates by geographical provenance (Canada and Europe) showed that 15 of the 27 European isolates (56%) harbored differences compared to 6 of the 126 Canadian isolates (5%). The fact that the European isolates were 10 times more likely to have differences in their repertoires was unexpected. Based on the proportions of the European and Canadian isolates and assuming that isolates from both provenances have an equal chance to have a missing plasmid, more variations should have been found among the Canadian isolates, which was clearly not the case.

**Table 1:** **Strains with an unusual repertoire of small plasmids**.

**Strain**	**Origin**	**Small plasmids analyzed^a^**				**TTSS**
		**pAsa1**	**pAsa2**	**pAsa3**	**pAsal1**	
A449	Europe	+	+	+	−	+
RS 534	Europe	+	+	+	−	−
JF3791	Europe	+	+	+	−	−
HER1084	Europe	+	+	+	−	−
HER1104	Europe	+	+	+	+^b^	−
JF2506	Europe	+	+	+	+p	+
JF2507	Europe	+	+	+	+p	+
JF2267	Europe	+	+	−	+	+
JF3517	Europe	+	+	−	+	+
JF3518	Europe	+	+	−	+	+
JF2869	Europe	+	+	−	+	−
JF4111	Europe	+	+	−	+	+
JF4112	Europe	+	+	−	+	+
JF4113	Europe	+	+	−	+	+
JF4114	Europe	+	+	−	+	+
RS 1835	Canada	+	+	+	−	+
SHY13-3795	Canada	+	+	+	−	+
SHY13-2317	Canada	+	+	−	+	+
SHY13-2425	Canada	+	+	−	+	+
SHY14-3502	Canada	+	+	−	+	+
SHY14-4161	Canada	+	+	−	+	+

a *Plasmids detected by PCR and restriction fragment profiling (+), by PCR but not by restriction fragment profiling (+p), or by neither PCR or restriction fragment profiling (−)*.

b *This isolate bears a pAsal1 variant named pAsal1B (Trudel et al., 2013)*.

There was also a significant difference in the propensity of each plasmid to cause a variation in the plasmidome. Our results showed that pAsa1 and pAsa2 are very stable, since all the isolates bore these two plasmids. This result partially contradicted the findings reported by Najimi et al. (2008), who claimed that pAsa2 may be less stable among the plasmidome than previously reported (Boyd et al., 2003). The finding by Najimi et al. may have been biased by the fact that only two isolates from turbots in Portugal were reported to have a missing pAsa2 and thus this statement would seem to be a local specificity (Najimi et al., 2008).

pAsa3 was missing for 12 isolates while pAsal1 was involved in three variants (including pAsal1B and two variants described in the Section pAsal1C and pAsal1D of the present study) and absent in six isolates. Compared to the strong propensity of the European isolates to have a missing plasmid (pAsa3 or pAsal1) from their plasmidomes, there was no robust geographical trend in terms of which plasmid was absent. A higher tendency to have a missing pAsa3 or pAsal1 has been observed in the past (Boyd et al., 2003; Najimi et al., 2008) but on smaller sets of isolates.

Interestingly, pAsa1 and pAsa3 are known to carry genes coding for a type II toxin-antitoxin (TA) system (Boyd et al., 2003). It was suggested that these TA systems might act as a post-segregational killing mechanism for daughter cells, which have not received the plasmids. Each plasmid encodes different TA system which is probably specific for their own maintenance in the cell (Boyd et al., 2003). Investigation using TAfinder (Shao et al., 2011) did not allow to find such systems for pAsa2 and pAsal1. The TA systems may help to explain the high and low level of stability among the plasmidome for pAsa1 and pAsal1, respectively. However, this does not give any clue concerning why pAsa2, which does not bears known TA system, is very stable and, at the opposite, why pAsa3, which has a TA system, is often missing of the plasmidome for many isolates. In fact, we cannot rule out the possibility that some clonal populations never acquired the missing plasmid (i.e., pAsa3 or pAsal1).

Unlike all the other small plasmids, pAsal1 bore an IS*AS11* (Fehr et al., 2006). This IS is activated by stressful conditions such as growth above 25°C and induces major rearrangements, including the loss of the TTSS, which is on the large plasmid pAsa5 (Daher et al., 2011; Tanaka et al., 2012). Tanaka et al. showed that stressful conditions may also induce the loss of pAsal1, most probably caused by its IS*AS11* (Tanaka et al., 2012). The propensity to lose this plasmid is coherent with the fact that no TA system was found on it. Given that a recombination and, consequently, the deletion of the TTSS region is an indication of the activation of IS*AS11*, we investigated the presence/absence of this locus in all isolates with a variable plasmidome by PCR genotyping (Table 1). Interestingly, while not systematic, we found a correlation between the loss of pAsal1 and the TTSS (Table S1). In fact, excluding the isolates containing pAsal1 variants (see next section), three of the four European isolates that had lost their pAsal1 had also lost their TTSS, with only A449 keeping its TTSS. The HER1104 isolate, which bears pAsal1B, had also lost its TTSS. On the other hand, none of the Canadian isolates, which miss their pAsal1, had lost their TTSS. In fact, it is possible to note that 11 European isolates had lost their TTSS (41%) compared to only five Canadian isolates (4%). This could be related to harsh culture conditions in the past promoting loss of TTSS region (Daher et al., 2011; Tanaka et al., 2012; Emond-Rheault et al., 2015b). Consequently, the increase absence of pAsa3 and pAsal1 in European isolates is maybe also related to that. However, since European isolates were collected from various fish species distinct to those of the Canadian isolates, we cannot exclude that host may also contribute to a selective pressure causing the stability or instability of the plasmidome.

**Figure 1:**
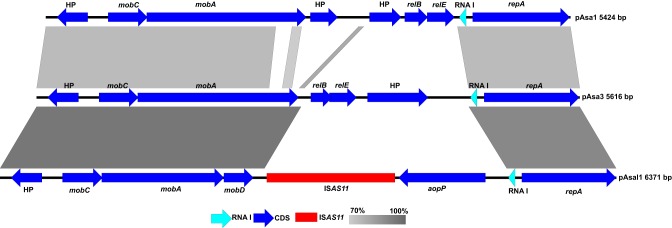
**Maps of the ColE2-type replicon plasmids pAsa1, pAsa3, and pAsal1**. The gray zones represent high identity regions of the sequences. The dark and light blue arrows represent coding sequences (CDSs) and the RNA I regulator, respectively. The red rectangle represents the IS*AS11* of pAsal1. HP means “hypothetical protein.” The complete map of pAsal1 is given in Figure S1.

*A. salmonicida* subsp. *salmonicida* isolates normally bear three ColE2-type replicon plasmids (pAsa1, pAsa3, and pAsal1). pAsa3 and pAsal1 share a high degree of identity (Figure 1; Trudel et al., 2013). This information takes its importance knowing that both pAsa3 and pAsal1 are less stable than pAsa1, which is another ColE2-type replicon plasmid. The main difference between pAsa3 and pAsal1 was the replacement of a gene coding for a hypothetical protein as well as the TA system by the IS*AS11* and the *aopP* gene in pAsal1.

### pAsal1C and pAsal1D

**Figure 2:**
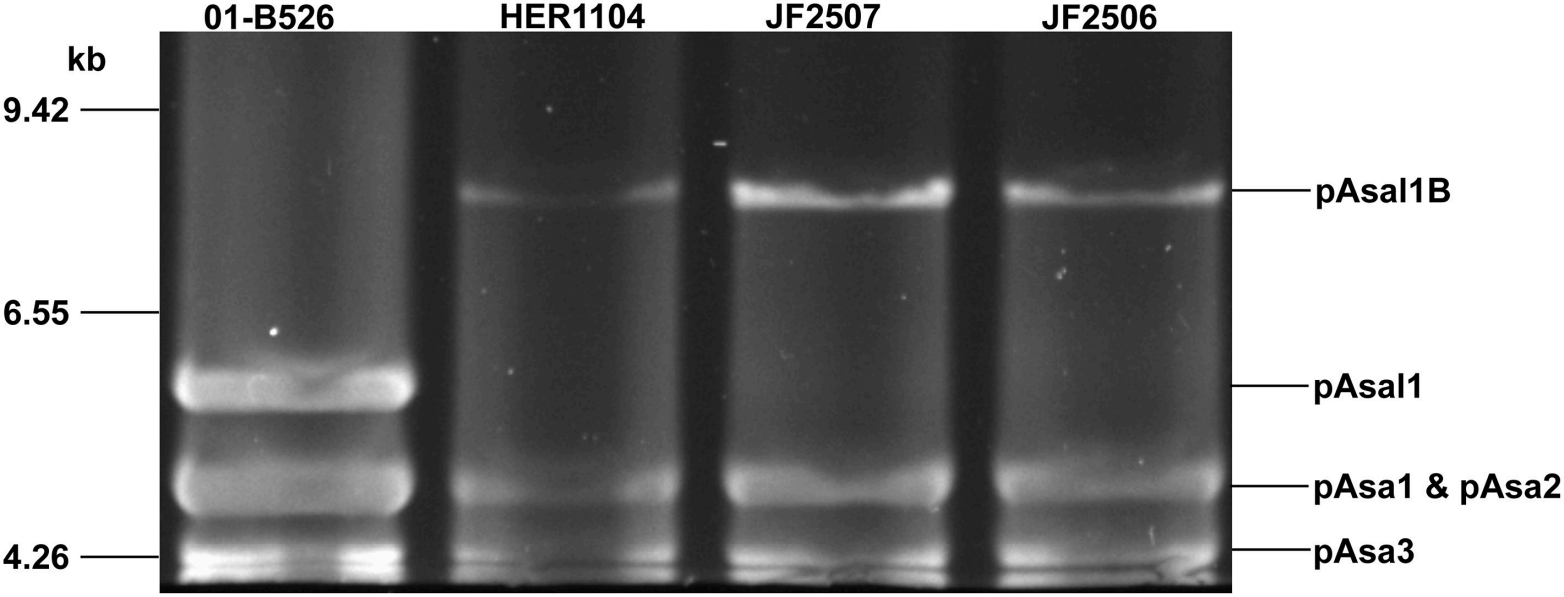
**Pulse field gel electrophoresis of digested small plasmids**. The purified small plasmids of isolates JF2506 and JF2507 were digested with *Eco*RI and were separated on 1% agarose gel by PFGE. HER1104 was also added since the bands were similar to those reported for the pAsal1 variant pAsal1B. Strain 01-B526 was used as a control of a normal plasmidome of small plasmids.

**Figure 3:**
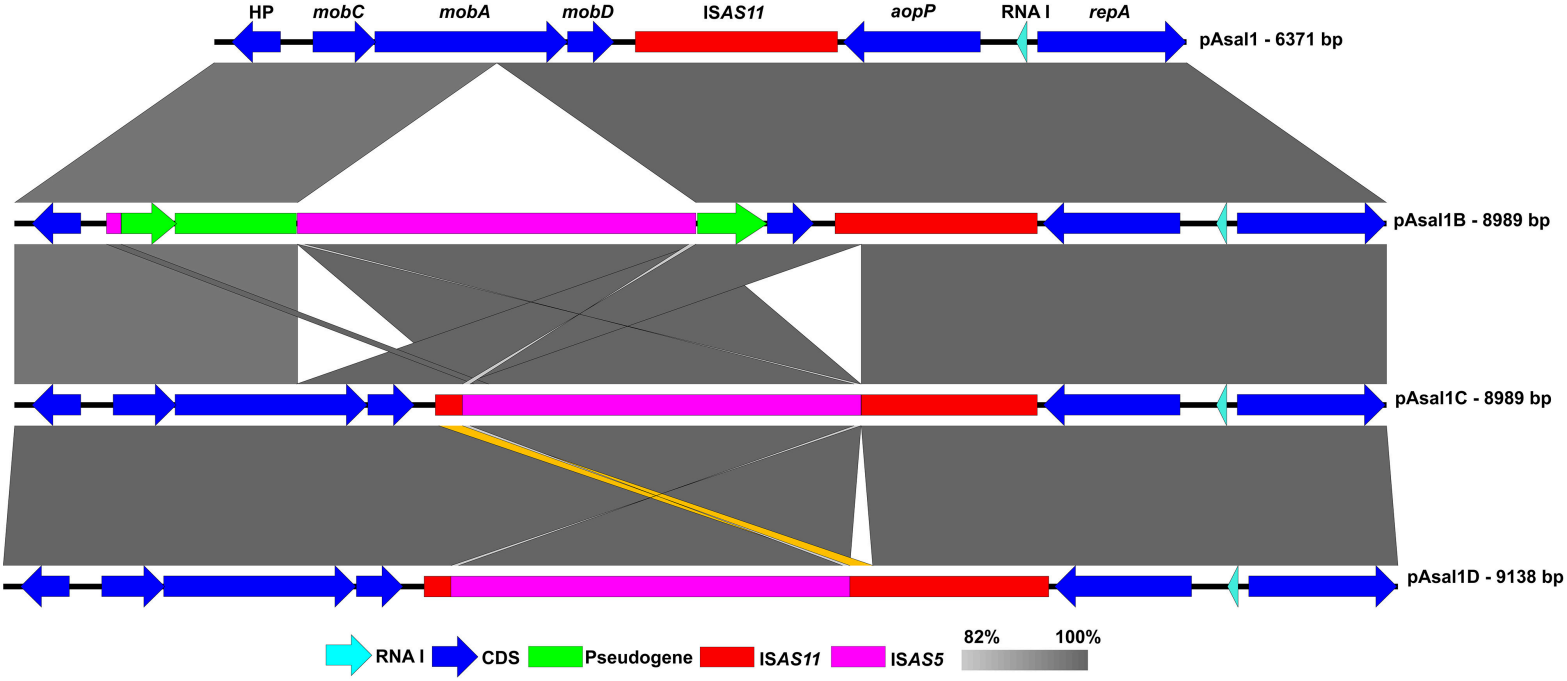
**Comparison of pAsal1 (JF2267), pAsal1B (HER1104), pAsa1C (JF2506), and pAsal1D (JF2507)**. The dark and light blue arrows represent coding sequences (CDSs) and the RNA I regulator, respectively. The purple and red rectangles represent IS*AS5* and IS*AS11*, respectively, while the green rectangles represent pseudogenes. The gray zones represent high identity regions of the sequences. The orange zone indicates the difference between pAsal1C and pAsal1D. The CDSs related to transposases and a 3′ part of the truncated *mobA* were not added for purposes of clarity. The complete map of pAsal1 is given in Figure S1.

pAsal1 was not observed on the agarose gels during the profiling of the JF2506 and JF2507 European isolates. However, a higher band like the one of the variant pAsal1B was visible for both isolates (Figure 2) and the gene *aopP* was detected by PCR. The small plasmids of the JF2506 and JF2507 isolates were investigated in greater depth by sequencing their DNA by NGS. The *de novo* assemblies revealed the presence of two variants that we named pAsal1C and pAsal1D (in JF2506 and JF2507, respectively). Interestingly, like the pAsal1B variant, both pAsal1C and pAsal1D bore an IS*AS5* (Figure 3). However, the IS*AS5*s of pAsal1B and pAsal1C-D were not inserted at the same position (Figure S1). According to the IS insertion mechanism, the recognition sequence (i.e., the target site) allowing the insertion by the transposase is duplicated on both sides of the IS (Berger and Haas, 2001). Given that the IS*AS5*s were not inserted at the same positions in pAsal1B and pAsal1C-D, we verified whether the recognition sequences were the same. The recognition sequences were 5′-AAGTT-3′ and 5′-TCACC-3′ for pAsal1B and pAsal1C-D, respectively. It is possible to rule out a mutation in one of the transposase genes as a cause of this difference in the recognition sequence since both IS*AS5*s shared 100% identity over 100% of their length. This prompted us to investigate the 12 complete IS*AS5*s in the chromosome and the large plasmids pAsa4 and pAsa5 of the A449 reference strain, which is the only isolate with a fully assembled chromosome, pAsa4 and pAsa5 sequences (Reith et al., 2008). The recognition sequences were mostly divergent, meaning that IS*AS5*s can transpose to many sites and can thus be considered as versatile. A homology search against the ISfinder database (Siguier et al., 2006) showed that IS*AS5* is a member of the IS*21* family. More than 100 IS*21* target sites have been sequenced, but no clear consensus has emerged (Berger and Haas, 2001). The present study confirmed the same observation for the IS*AS5* in *A. salmonicida* subsp. *salmonicida*.

Even if the IS*AS5* is inserted at the same site in the IS*AS11* of pAsal1C and pAsal1D, the two plasmids diverge in the region around this IS. There is a duplication of a portion of IS*AS11* in pAsa1D but not in pAsal1C (Figure 3). The difference is subtle but important given that the transposase is complete in pAsal1D (404 a.a.) while it is truncated in pAsal1C (374 a.a.), meaning that the pAsal1D transposase may still recognize the inverted-repeat sequences of IS*AS11* and thus promote the transposition of IS*AS11*, which includes IS*AS5*, to generate a 4093-bp IS.

Of the 153 isolates analyzed, none of the three variants (pAsa1B-C-D) was found twice, meaning that each variant might be a unique event. However, the IS*AS5*s in pAsal1C and pAsal1D were inserted at the same position but only gave rise to a subtle difference. A clonal strain that underwent an unknown event that led to the difference between both plasmids can be ruled out since JF2506 and JF2507 were isolated in Norway and Scotland, respectively (Burr and Frey, 2007; Studer et al., 2013). If this were the case, it would mean that there is a very small population of strains with pAsal1C-D-like plasmids. It is more logical to propose that pAsal1C and its homolog pAsal1D are the products of convergent evolution since the insertion of an IS in another IS (as it is the case here) would minimize possible deleterious effects, unlike an insertion in a gene. However, why these plasmid variants, including pAsal1B, predominated over the wild-type plasmid pAsal1 remains to be investigated, especially given that a large IS such as IS*AS5* is likely to cause a major metabolic burden (pAsal1-B-C-D variants are ~42% larger than pAsal1). Nevertheless, the IS*AS5* in pAsal1C and pAsal1D does not seems to impact on the virulence of the isolates knowing that both were isolated from infected Atlantic salmons (Burr and Frey, 2007; Studer et al., 2013).

As observed in another study (Tanaka et al., 2012) and suggested in the present study, stressful conditions may induce a loss of pAsal1, likely caused by the activation of IS*AS11*. If two populations of pAsal1 exist, one wild-type and one variant with a disrupted IS*AS11* (such as pAsal1C and pAsal1D), a stressful condition will have less or no effect on the variant since its IS*AS11* cannot be activated, and the variant will predominate over the wild-type plasmid. Knowing that pAsal1-like plasmids bear *aopP*, which encodes a TTSS effector, strains with a pAsal1 variant have a greater chance of remaining virulent following a stress if one assumes that the TTSS region is still present in pAsa5. In the case of pAsal1B, in which the IS*AS5* is inserted in *mobA* and not in IS*AS11*, it is possible that a simple insertion of IS*AS5* prevents the loss of the plasmid in stressful conditions. This possibility is reinforced by the fact that the TTSS on pAsa5 was lost (Table 1) in the strain bearing pAsal1B. Unlike pAsa5, for which the mechanism regulating the recombination of two IS*AS11*s is known (Tanaka et al., 2012), the mechanism responsible for the loss of pAsal1 is not known, and it would be perilous to suggest an explication here.

### Other plasmids

Isolate 2004-208 was shown to be positive for pAsa3 by PCR in another study but has an unusual gel migration profile (Trudel et al., 2013). Sequencing by NGS and *de novo* assembly showed that this isolate does indeed possess a pAsa3-like plasmid. However, we suspect that another plasmid (pSBAL6780-like) sharing sequence identity with a plasmid in *Shewanella baltica* OS678 (Caro-Quintero et al., 2012) is present in 2004-208 and interferes with the *de novo* assembly of pAsa3. Further investigations will be required to confirm this since it is currently impossible to rigorously assemble the pAsa3 and the pSBAL6780-like plasmids. Large repeated elements may be responsible for the inability to determine the complete sequences. Using third-generation sequencing to generate long reads (>7 kb) may be the solution for overcoming this problem (Koren et al., 2013). Lastly, a new small plasmid in JF3791 was detected on agarose gels and confirmed by *de novo* assembly. However, since this plasmid is not a part of the normal plasmidome, we did not characterize it here.

### Copy number

pAsa1, pAsa3, and pAsal1 (and its derivatives) are ColE2-type replicons and pAsa2 is a ColE1-type replicon. These two types of replicons are usually found in high copy numbers in cells (Lilly and Camps, 2015). Based on our results, we investigated the average copy per chromosome (that we estimated to be in a single copy per cell) which was previously estimated at approximately 55 copy per cell (Hackett et al., 1984). We used the sequencing reads as indicated in the Materials and Methods Section and found significant variations among the total number of small plasmids per cell, which ranged from ~32 for M19878-11 to ~326 for 2010-47K18 (Figure S2). However, we cannot rule out the possibility that this large variation in the number of copies might be caused by the biological state of the cells at the time of the DNA extraction and/or by the DNA extraction procedure itself. Despite the large variation, only 2010-47K18 isolate could be considered as an outlier for pAsal1, pAsa2, and pAsa3 (Figure S3).

**Figure 4:**
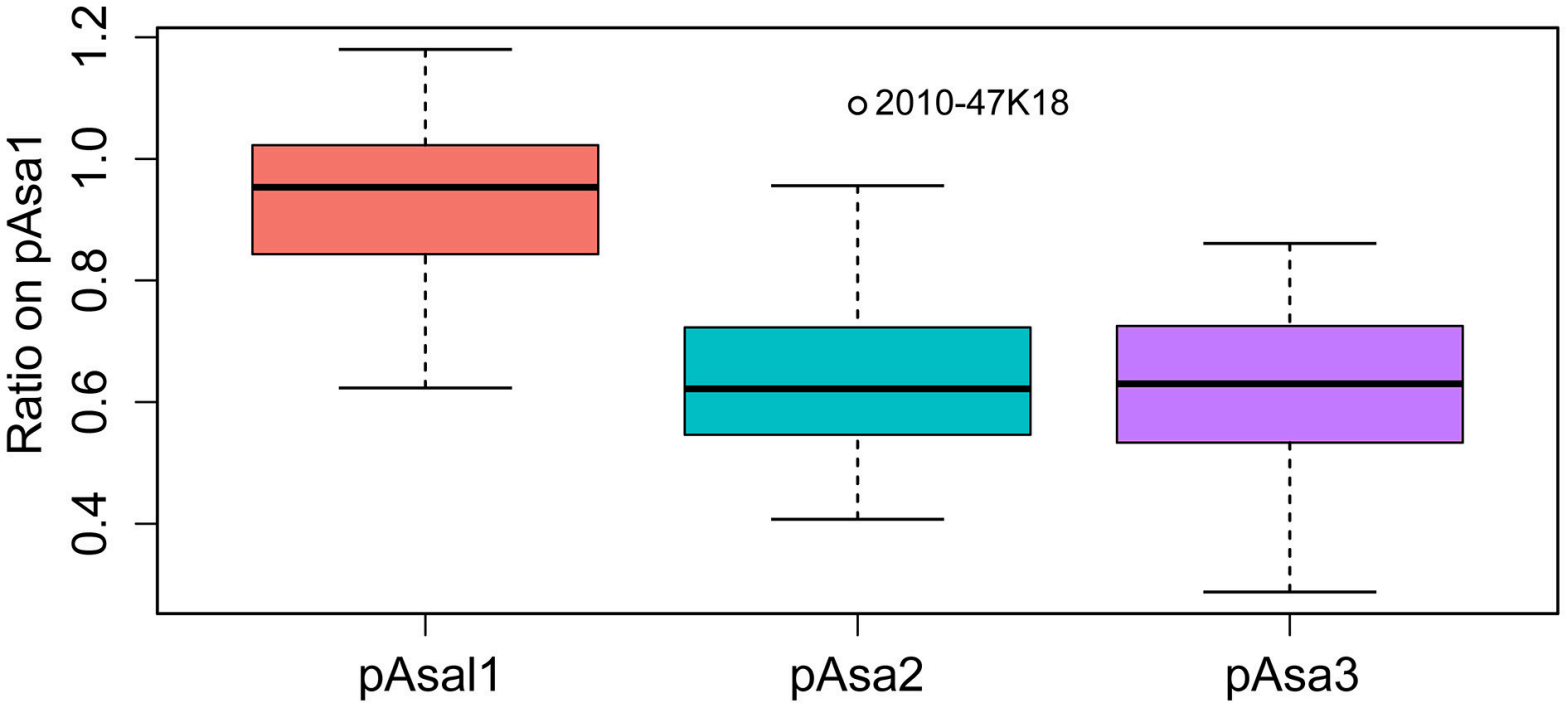
**Boxplot analysis of the plasmid copy number ratios relative to pAsa1**. This analysis included small plasmids for all the isolates for which the DNA was sequenced and which are listed in Table S3.

Even if the high variation might be partially engendered by bias as indicated above, it was possible to determine that the relative proportion of each plasmid in the isolates appeared to be constant. To confirm this, the copy number ratios of pAsa2, pAsa3, and pAsal1 on pAsa1 (which is present in all the isolates) were computed for all the sequenced isolates. A boxplot analysis of the dataset revealed that pAsa1 is clearly present in the highest number per cell, followed closely by pAsal1, and then pAsa2 and pAsa3, which were almost always present in approximately the same copy number per cell (Figure 4).

### Sequence differences among pAsa1, pAsa2, pAsa3, and pAsal1

As mentioned above, the JF2506 and JF2507 isolates harbored the pAsal1C and pAsal1D variants of pAsal1, respectively. Smaller differences (SNPs and InDels) were also investigated in the three cryptic plasmids in addition to all the pAsal1s (Table S3). Interestingly, of the 27 pAsa1s analyzed, only the one from JF3791 had a SNP (transition) in an intergenic region. For pAsa2, 01-B526, and JF3517 had a SNP (transition) in an intergenic region while RS 1752 had a 43-bp duplication in an intergenic region. pAsa3 exhibited the most mutations, many of which were non-synonymous, and one of which was a small 16-bp deletion in *mobA* and *mobB* that disrupted their ORFs. For the pAsal1 plasmids, the reference strain JF2267 and the M13566-12 isolate had two SNPs and a 184-bp deletion, respectively, in *mobA*. Almost all the pAsal1 assemblies had a break in IS*AS11*, which was expected given that ISs are one of the main causes of contig breaks during *de novo* assemblies of this bacterium (Vincent et al., 2014a, 2015). To investigate this IS, PCR amplifications were performed for the IS*AS11*s of the sequenced isolates (Figure S4). All the amplified IS*AS11*s had a band at the expected size of 1700 bp. The PCR products of the IS*AS11*s in the pAsal1C and pAsalD variants in JF2506 and JF2507, respectively, had higher molecular weights, as expected. A striking observation was that the RS 534, JF3791, and JF2507 isolates also produced a 1700-bp band, albeit fainter, even though the two first isolates are not thought to harbor pAsal1 and the third is known to harbor pAsal1D. This might indicate that the cells possess a low copy number of pAsal1 or that a small sub-population of cells bears pAsal1. Less than five sequencing reads were mapped on *aopP* for RS 534 and JF3791. However, since only a couple colonies were used to extract the sequenced DNA, it is possible that the sub-population harboring pAsal1 was missed.

### The mobilization genes

As stated elsewhere, the pAsa1-2-3 small plasmids present in *A. salmonicida* subsp. *salmonicida* carry the genes *mobABCD*, which are related to mobilization, but lack those for transfer (Boyd et al., 2003). Also stated in the same article, two large plasmids in the reference strain A449 possess genes related to transfer (Boyd et al., 2003). This was confirmed by the sequencing of the two large plasmids: pAsa4 and pAsa5 (Reith et al., 2008; Piotrowska and Popowska, 2015). This let suppose that transfer proteins encoded by these large plasmids may act in *trans* for the transfer of the small plasmids. However, the situation is less clear for pAsal1.

A striking observation was that *mobA* was a predilection target for IS insertions (pAsal1B), deletions, and mutations. MobA, the product of the *mobA* gene, is divided into two functional domains: a relaxase (N-terminal) and a primase (C-terminal). Interestingly, all the pAsal1s harbored a truncated *mobA* (Figure S1). Since the MobAs of pAsal1 and its derivatives likely have a complete N-terminal section (420 a.a.) and since it has been shown that a disrupted MobA may still possess relaxase activity (Becker and Meyer, 2002), it is possible that this activity is also present in the MobA of pAsal1, even if truncated. However, transcriptomic and proteomic analyses would be required to confirm that one or both of the domains are still produced and are stable.

*mobB* encodes a protein with a C-terminal membrane domain and an N-terminal domain that interact with the primase (MobA) and increase the transfer frequency of the plasmid (Meyer, 2011). Since *mobB* overlaps *mobA* (translated in different frames), it is also truncated in pAsal1 (Figure S1), and since the C-terminal is crucial for anchoring the membrane, the resulting truncated protein is likely to be inactive. Given that pAsa3 harbors complete *mobA* and *mobB* genes and is homologous to pAsal1 (Figure 1), if the products of pAsal1 are inactive, those of pAsa3 may act for pAsal1 *in trans*. Given all the above, the mobilization of pAsal1 will be important to study knowing that this plasmid encodes a TTSS effector (Fehr et al., 2006).

## Conclusions

The plasmidome of 153 *A. salmonicida* subsp. *salmonicida* isolates were analyzed by PCR genotyping and restriction fragment profiling. In addition, 26 isolates were studied by NGS. These analyses shed light on an unsuspected dichotomy between European and Canadian isolates with respect to their propensity to have a missing pAsa3 and pAsal1, two homologous ColE2-type replicons, among their plasmidome. These two plasmids were also more prone to mutations in their sequences than pAsa1 and pAsa2. The mobilization genes were the main targets of these mutations. However, despite the different geographical origins of the isolates, their average copy number ratios were the same. Two new variants of pAsal1 (pAsal1C and pAsal1D) were found in European isolates, and the sequencing of their DNA revealed the presence of an IS*AS5*, which disrupted the IS*AS11*. The reasons for the predominance of the variants over the wild-type pAsal1 are unknown, but the disruption of the IS*AS11* may have made the plasmids less sensitive to stressful conditions. While there was a diverse array of small plasmids in the isolates, there appeared to be conservative pressure on these plasmids despite the fact that pAsa1, pAsa2, and pAsa3 were cryptic. We showed that there is a close evolutionary link between the cryptic plasmid pAsa3 and the non-cryptic pAsal1, indicating that small plasmids may be templates for the evolution of *A. salmonicida* subsp. *salmonicida* knowing that pAsal1 is no longer a cryptic plasmid since it encodes a TTSS effector. Moreover, even pAsal1 continues to be a template for evolutionary innovations with three known variants: pAsal1B-C-D. Another interesting observation in the same way, is the discovery in *A. sobria* and *A. hydrophila* of small plasmids (pAQ2-1 and pAQ2-2) homologs to those found in *A. salmonicida*, but bearing a quinolone resistance gene (*qnrS2*) (Han et al., 2012). Finally, this study revealed not only extensive homogeneity but also widespread diversity among ColE1- and ColE2-type replicons in *A. salmonicida* subsp. *salmonicida* in part linked with the geographical origin of the tested isolates.

## Author contributions

SA, AV, and SC designed the research protocol; SA, AV, MT, and RC performed the research; SA, AV, MT, and RC analyzed the data; and SA, AV, and SC wrote the paper.

## Conflict of interest statement

The authors declare that the research was conducted in the absence of any commercial or financial relationships that could be construed as a potential conflict of interest.

## Supplementary material

Table S1 ***A*. *salmonicida* isolates analyzed**.

Table S2 **Primers used for the PCR genotyping**.

Table S3 **Sequence differences (SNPs and InDels) found in the small plasmids of the sequenced strains of *A*. *salmonicida***.

Figure S1 **Map of the reference pAsal1 plasmid and the insertion sites of the IS*AS5*s in pAsal1B, pAsal1C, and pAsal1D**. The arrows in dark and light blue represent the coding sequences and the RNA I regulator, respectively. The red and green rectangles represent the IS*AS11* and the putative pseudogene *mobB*, respectively. The insertion sites (and their sequences) for the IS*AS5*s in all pAsal1B-C-Ds are shown.

Figure S2 **Average copy numbers of pAsal1, pAsa1, pAsa2, and pAsa3 for all the isolates for which the DNA was sequenced**. The copy numbers for IS*AS5* and IS*AS11* were also computed since they are found on pAsal1 (and its variants for IS*AS5*). As indicated in the main article, the coverages provided by the sequencing reads were used to infer the information on the average copy number for each plasmid and IS.

Figure S3 **Boxplots of the average copy number of each plasmid found in the strains presented in Figure S2**.

Figure S4 **PCR amplifications of the IS*AS11* in pAsal1 of all 26 strains for which the genomic DNA was sequenced**.

## References

[B1] Adams C. A. Austin B. Meaden P. G. McIntosh D. (1998). Molecular characterization of plasmid-mediated oxytetracycline resistance in *Aeromonas salmonicida*. Appl. Environ. Microbiol. 64, 4194–4201. 9797265 10.1128/aem.64.11.4194-4201.1998 PMC106627

[B2] Becker E. C. Meyer R. J. (2002). MobA, the DNA strand transferase of plasmid R1162. The minimal domain required for DNA processing at the origin of transfer. J. Biol. Chem. 277, 14575–14580. 10.1074/jbc.M110759200 11839744

[B3] Belland R. J. Trust T. J. (1988). DNA:DNA reassociation analysis of *Aeromonas salmonicida*. J. Gen. Microbiol. 134, 307–315. 10.1099/00221287-134-2-307 3171541

[B4] Belland R. J. Trust T. J. (1989). *Aeromonas salmonicida* plasmids: plasmid-directed synthesis of proteins *in vitro* and in *Escherichia coli* minicells. Microbiology 135, 513–524. 10.1099/00221287-135-3-513

[B5] Berger B. Haas D. (2001). Transposase and cointegrase: specialized transposition proteins of the bacterial insertion sequence IS*21* and related elements. Cell. Mol. Life Sci. 58, 403–419. 10.1007/PL00000866 11315188 PMC11337337

[B6] Bolger A. M. Lohse M. Usadel B. (2014). Trimmomatic: a flexible trimmer for Illumina sequence data. Bioinformatics 30, 2114–2120. 10.1093/bioinformatics/btu170 24695404 PMC4103590

[B7] Boyd J. Williams J. Curtis B. Kozera C. Singh R. Reith M. (2003). Three small, cryptic plasmids from *Aeromonas salmonicida* subsp. *salmonicida* A449. Plasmid 50, 131–144. 10.1016/S0147-619X(03)00058-1 12932739

[B8] Burr S. E. Frey J. (2007). Analysis of type III effector genes in typical and atypical *Aeromonas salmonicida*. J. Fish Dis. 30, 711–714. 10.1111/j.1365-2761.2007.00859.x 17958615

[B9] Caro-Quintero A. Auchtung J. Deng J. Brettar I. Höfle M. Tiedje J. M.. (2012). Genome sequencing of five *Shewanella baltica* strains recovered from the oxic-anoxic interface of the baltic sea. J. Bacteriol. 194, 1236–1236. 10.1128/JB.06468-11 22328742 PMC3294791

[B10] Charette S. J. Brochu F. Boyle B. Filion G. Tanaka K. H. Derome N. (2012). Draft genome sequence of the virulent strain 01-B526 of the fish pathogen Aeromonas salmonicida. J. Bacteriol. 194, 722–723. 10.1128/JB.06276-11 22247525 PMC3264088

[B11] Charette S. J. Cosson P. (2004). Preparation of genomic DNA from *Dictyostelium discoideum* for PCR analysis. Biotechniques 36, 574–575. 10.2144/3604A0574 15088372

[B12] Coil D. Jospin G. Darling A. E. (2014). A5-miseq: an updated pipeline to assemble microbial genomes from Illumina MiSeq data. Bioinformatics 31, 587–589. 10.1093/bioinformatics/btu661 25338718

[B13] Daher R. K. Filion G. Tan S. G. E. Dallaire-Dufresne S. Paquet V. E. Charette S. J. (2011). Alteration of virulence factors and rearrangement of pAsa5 plasmid caused by the growth of *Aeromonas salmonicida* in stressful conditions. Vet. Microbiol. 152, 353–360. 10.1016/j.vetmic.2011.04.034 21621930

[B14] Dallaire-Dufresne S. Tanaka K. H. Trudel M. V. Lafaille A. Charette S. J. (2014). Virulence, genomic features, and plasticity of *Aeromonas salmonicida* subsp. salmonicida, the causative agent of fish furunculosis. Vet. Microbiol. 169, 1–7. 10.1016/j.vetmic.2013.06.025 23890675

[B15] Dautremepuits C. Fortier M. Croisetiere S. Belhumeur P. Fournier M. (2006). Modulation of juvenile brook trout (*Salvelinus fontinalis*) cellular immune system after *Aeromonas salmonicida* challenge. Vet. Immunol. Immunopathol. 110, 27–36. 10.1016/j.vetimm.2005.09.008 16263179

[B16] Emond-Rheault J.-G. Vincent A. T. Trudel M. V. Brochu F. Tanaka K. H. Attéré S. A.. (2015a). Variants of a genomic island in *Aeromonas salmonicida* subsp. salmonicida link isolates with their geographical origins. Vet. Microbiol. 175, 68–76. 10.1016/j.vetmic.2014.11.014 25480167

[B17] Emond-Rheault J.-G. Vincent A. T. Trudel M. V. Frey J. Frenette M. Charette S. J. (2015b). AsaGEI2b: a new variant of a genomic island identified in the *Aeromonas salmonicida* subsp. *salmonicida* JF3224 strain isolated from a wild fish in Switzerland. FEMS Microbiol. Lett. 362:fnv093. 10.1093/femsle/fnv093 26048417

[B18] Fehr D. Casanova C. Liverman A. Blazkova H. Orth K. Dobbelaere D.. (2006). AopP, a type III effector protein of *Aeromonas salmonicida*, inhibits the NF-kappaB signalling pathway. Microbiology 152, 2809–2818. 10.1099/mic.0.28889-0 16946275

[B19] García J. A. Larsen J. L. Dalsgaard I. Pedersen K. (2000). Pulsed-field gel electrophoresis analysis of *Aeromonas salmonicida* ssp. salmonicida. FEMS Microbiol. Lett. 190, 163–166. 10.1016/S0378-1097(00)00322-0 10981708

[B20] García-Alcalde F. Okonechnikov K. Carbonell J. Cruz L. M. Götz S. Tarazona S.. (2012). Qualimap: evaluating next-generation sequencing alignment data. Bioinformatics 28, 2678–2679. 10.1093/bioinformatics/bts503 22914218

[B21] Hackett J. L. Lynch W. H. Paterson W. D. Coombs D. H. (1984). Extracellular protease, extracellular haemolysin, and virulence in *Aeromonas salmonicida*. Can. J. Fish. Aquat. Sci. 41, 1354–1360. 10.1139/f84-166

[B22] Han J. E. Kim J. H. Choresca J. H. Jr. Shin S. P. Jun J. W. Chai J. Y.. (2012). First description of ColE-type plasmid in *Aeromonas* spp. carrying quinolone resistance (*qnrS2*) gene. Lett. Appl. Microbiol. 55, 290–294. 10.1111/j.1472-765X.2012.03293.x 22862417

[B23] Hänninen M. L. Ridell J. Hirvelä-Koski V. (1995). Phenotypic and molecular characteristics of *Aeromonas salmonicida* subsp. salmonicida isolated in southern and northern Finland. J. Appl. Bacteriol. 79, 12–21. 10.1111/j.1365-2672.1995.tb03118.x 7665387

[B24] Koren S. Harhay G. P. Smith T. P. L. Bono J. L. Harhay D. M. Mcvey S. D.. (2013). Reducing assembly complexity of microbial genomes with single-molecule sequencing. Genome Biol. 14:R101. 10.1186/gb-2013-14-9-r101 24034426 PMC4053942

[B25] L'Abée-Lund T. M. Sørum H. (2002). A global non-conjugative Tet C plasmid, pRAS3, from *Aeromonas salmonicida*. Plasmid 47, 172–181. 10.1016/S0147-619X(02)00001-X 12151232

[B26] Lilly J. Camps M. (2015). Mechanisms of theta plasmid replication. Microbiol. Spectr. 3:PLAS-0029-2014. 10.1128/microbiolspec.plas-0029-2014 26104556 PMC4441207

[B27] Liu Y. Popp B. Schmidt B. (2014). CUSHAW3: sensitive and accurate base-space and color-space short-read alignment with hybrid seeding. PLoS ONE 9:e86869. 10.1371/journal.pone.0086869 24466273 PMC3899341

[B28] Meyer R. (2011). Functional organization of MobB, a small protein required for efficient conjugal transfer of plasmid R1162. J. Bacteriol. 193, 3904–3911. 10.1128/JB.05084-11 21622757 PMC3147529

[B29] Najimi M. Lemos M. L. Osorio C. R. (2008). Distribution of small plasmids in *Aeromonas salmonicida* subsp. salmonicida strains isolated from NW Spain and Portugal: evidence of clonality in strains isolated from turbot, Psetta maxima (L.). J. Fish Dis. 31, 469–472. 10.1111/j.1365-2761.2007.00896.x 18471103

[B30] O'hIci B. Olivier G. Powell R. (2000). Genetic diversity of the fish pathogen *Aeromonas salmonicida* demonstrated by random amplified polymorphic DNA and pulsed-field gel electrophoresis analyses. Dis. Aquat. Organ. 39, 109–119. 10.3354/dao039109 10715816

[B31] Pearson W. R. Lipman D. J. (1988). Improved tools for biological sequence comparison. Proc. Natl. Acad. Sci. U.S.A. 85, 2444–2448. 10.1073/pnas.85.8.2444 3162770 PMC280013

[B32] Piotrowska M. Popowska M. (2015). Insight into the mobilome of Aeromonas strains. Front. Microbiol. 6:494. 10.3389/fmicb.2015.00494 26074893 PMC4444841

[B33] Reith M. E. Singh R. K. Curtis B. Boyd J. M. Bouevitch A. Kimball J.. (2008). The genome of *Aeromonas salmonicida* subsp. salmonicida A449: insights into the evolution of a fish pathogen. BMC Genomics 9:427. 10.1186/1471-2164-9-427 18801193 PMC2556355

[B34] Rice P. Longden I. Bleasby A. (2000). EMBOSS: the european molecular biology open software suite. Trends Genet. 16, 276–277. 10.1016/S0168-9525(00)02024-2 10827456

[B35] Rutherford K. Parkhill J. Crook J. Horsnell T. Rice P. Rajandream M. A.. (2000). Artemis: sequence visualization and annotation. Bioinformatics 16, 944–945. 10.1093/bioinformatics/16.10.944 11120685

[B36] Shao Y. Harrison E. M. Bi D. Tai C. He X. Ou H. Y.. (2011). TADB: A web-based resource for Type 2 toxin-antitoxin loci in bacteria and archaea. Nucleic Acids Res. 39, D606–D611. 10.1093/nar/gkq908 20929871 PMC3013778

[B37] Siguier P. Perochon J. Lestrade L. Mahillon J. Chandler M. (2006). ISfinder: the reference centre for bacterial insertion sequences. Nucleic Acids Res. 34, D32–D36. 10.1093/nar/gkj014 16381877 PMC1347377

[B38] Sørum H. L'Abée-Lund T. M. Solberg A. Wold A. (2003). Integron-Containing IncU R Plasmids pRAS1 and pAr-32 from the Fish Pathogen *Aeromonas salmonicida*. Antimicrob. Agents Chemother. 47, 1285–1290. 10.1128/AAC.47.4.1285-1290.2003 12654659 PMC152485

[B39] Studer N. Frey J. Vanden Bergh P. (2013). Clustering subspecies of *Aeromonas salmonicida* using IS*630* typing. BMC Microbiol. 13:36. 10.1186/1471-2180-13-36 23406017 PMC3608246

[B40] Sullivan M. J. Petty N. K. Beatson S. A. (2011). Easyfig: a genome comparison visualiser. Bioinformatics 27, 1009–1010. 10.1093/bioinformatics/btr039 21278367 PMC3065679

[B41] Tanaka K. H. Dallaire-Dufresne S. Daher R. K. Frenette M. Charette S. J. (2012). An insertion sequence-dependent plasmid rearrangement in *Aeromonas salmonicida* causes the loss of the type three secretion system. PLoS ONE 7:e33725. 10.1371/journal.pone.0033725 22432045 PMC3303853

[B42] R Core Team (2013). R: A Language and Environment for Statistical Computing. Vienna: R Found. Stat. Comput.

[B43] Trudel M. V. Tanaka K. H. Filion G. Daher R. K. Frenette M. Charette S. J. (2013). Insertion sequence *AS5* (IS*AS5*) is involved in the genomic plasticity of *Aeromonas salmonicida*. Mob. Genet. Elements 5, 1–7. 10.4161/mge.25640 PMC3742599 23956951

[B44] Vincent A. T. Boyle B. Derome N. Charette S. J. (2014a). Improvement in the DNA sequencing of genomes bearing long repeated elements. J. Microbiol. Methods 107, 186–188. 10.1016/j.mimet.2014.10.016 25447886

[B45] Vincent A. T. Tanaka K. H. Trudel M. V. Frenette M. Derome N. Charette S. J. (2015). Draft genome sequences of two *Aeromonas salmonicida* subsp. salmonicida isolates harboring plasmids conferring antibiotic resistance. FEMS Microbiol. Lett. 362, 1–4. 10.1093/femsle/fnv002 25724776

[B46] Vincent A. T. Trudel M. V. Paquet V. E. Boyle B. Tanaka K. H. Dallaire-Dufresne S.. (2014b). Detection of variants of the pRAS3, pAB5S9, and pSN254 plasmids in *Aeromonas salmonicida* subsp. salmonicida: multidrug-resistance, interspecies exchanges, and plasmid reshaping. Antimicrob. Agents Chemother. 58, 7367–7374. 10.1128/AAC.03730-14 25267667 PMC4249516

