# Supplementary material for: Diversity and Homogeneity among Small Plasmids of Aeromonas salmonicida subsp. salmonicida Linked with Geographical Origin
Source: Front Microbiol. 2015 Nov 23;6:1274. doi: 10.3389/fmicb.2015.01274 (PMC4655240; doi:10.3389/fmicb.2015.01274)
Supplement: Table S1 — A. salmonicida isolates analyzed. [file Table1.PDF]

Table S1. ***A. salmonicida*** **isolates analyzed**

Isolate[a]	Source (host)	Origin[b]	TTSS[c]	Plasmid present[d]				Reference
				pAsa1	pAsa2	pAsa3	pAsal1	
2009-178 K9	Atlantic salmon	Canada, NB	+	+	+	+	+	(Trudel *et al.*, 2013)
2009-157 K5	Brook trout	Canada, NB	+	+	+	+	+	(Trudel *et al.*, 2013)
2010-47 K18	Brook trout	Canada, NB	+	+	+	+	+	(Trudel *et al.*, 2013)
2004-05 MF26	-	Canada, NB	+	+	+	+	+	(Trudel *et al.*, 2013)
2009-195 K29	Brook trout	Canada, NB	+	+	+	+	+	(Trudel *et al.*, 2013)
2005-70	-	Canada, NB	+	+	+	+	+	(Trudel *et al.*, 2013)
2009-144 K3	Brook trout	Canada, NB	+	+	+	+	+	(Trudel *et al.*, 2013)
2005-175 K2	Brook trout	Canada, NB	+	+	+	+	+	(Trudel *et al.*, 2013)
2004-208	-	Canada, NB	+	+	+	+	+	(Trudel *et al.*, 2013)
2004-68 K52	Atlantic salmon	Canada, NS	+	+	+	+	+	(Trudel *et al.*, 2013)
RS 1458	Rainbow trout	Canada, On	+	+	+	+	+	This study
RS 1706	Chinook salmon	Canada, On	+	+	+	+	+	This study
RS 1835	Coho salmon	Canada, On	+	+	+	+	-	This study
RS 1752	Pumpkinseed	Canada, On	+	+	+	+	+	This study
RS 1705	Brook trout	Canada, On	+	+	+	+	+	This study
RS 1744	Coho salmon	Canada, On	+	+	+	+	+	This study
01-B522	Brook trout	Canada, Qc	+	+	+	+	+	(Trudel *et al.*, 2013)
01-B526	Brook trout	Canada, Qc	+	+	+	+	+	(Trudel *et al.*, 2013)
01-B516	Brook trout	Canada, Qc	+	+	+	+	+	(Trudel *et al.*, 2013)
07-9324	Brook trout	Canada, Qc	+	+	+	+	+	(Trudel *et al.*, 2013)
07-7817	-	Canada, Qc	+	+	+	+	+	(Trudel *et al.*, 2013)
07-7346	Atlantic salmon	Canada, Qc	+	+	+	+	+	(Trudel *et al.*, 2013)
07-5957	Atlantic salmon	Canada, Qc	-	+	+	+	+	(Trudel *et al.*, 2013)
08-2647	Brook trout	Canada, Qc	+	+	+	+	+	(Trudel *et al.*, 2013)
09-0167	Atlantic salmon	Canada, Qc	+	+	+	+	+	(Trudel *et al.*, 2013)
07-7287	Brook trout	Canada, Qc	-	+	+	+	+	(Trudel *et al.*, 2013)
08-2783	Brook trout	Canada, Qc	+	+	+	+	+	(Trudel *et al.*, 2013)
08-4188	Brook trout	Canada, Qc	+	+	+	+	+	(Trudel *et al.*, 2013)
5093-3	Brook trout	Canada, Qc	+	+	+	+	+	(Trudel *et al.*, 2013)
5093-4	Brook trout	Canada, Qc	+	+	+	+	+	(Trudel *et al.*, 2013)
5490-1	Brook trout	Canada, Qc	+	+	+	+	+	(Trudel *et al.*, 2013)
5490-2	Brook trout	Canada, Qc	+	+	+	+	+	(Trudel *et al.*, 2013)
5490-4	Brook trout	Canada, Qc	+	+	+	+	+	(Trudel *et al.*, 2013)
5490-5	Brook trout	Canada, Qc	+	+	+	+	+	(Trudel *et al.*, 2013)
5704-1	Brook trout	Canada, Qc	+	+	+	+	+	(Trudel *et al.*, 2013)
5704-2	Brook trout	Canada, Qc	+	+	+	+	+	(Trudel *et al.*, 2013)
5704-4	Brook trout	Canada, Qc	+	+	+	+	+	(Trudel *et al.*, 2013)

5704-5	Brook trout	Canada, Qc	+	+	+	+	+	(Trudel *et al.*, 2013)
5704-6	Brook trout	Canada, Qc	+	+	+	+	+	(Trudel *et al.*, 2013)
5704-3	Brook trout	Canada, Qc	+	+	+	+	+	(Trudel *et al.*, 2013)
M17524-09	Brook trout	Canada, Qc	-	+	+	+	+	(Trudel *et al.*, 2013)
M14349-09	Atlantic salmon	Canada, Qc	-	+	+	+	+	(Trudel *et al.*, 2013)
M23281-09	Brook trout	Canada, Qc	+	+	+	+	+	(Trudel *et al.*, 2013)
M23067-09	Brook trout	Canada, Qc	+	+	+	+	+	(Trudel *et al.*, 2013)
M19438-09	Brook trout	Canada, Qc	+	+	+	+	+	(Trudel *et al.*, 2013)
M16583-09	Brook trout	Canada, Qc	+	+	+	+	+	(Trudel *et al.*, 2013)
M14231-09	Atlantic salmon	Canada, Qc	-	+	+	+	+	(Trudel *et al.*, 2013)
M11743-09	Brook trout	Canada, Qc	+	+	+	+	+	(Trudel *et al.*, 2013)
M11431-09	Brook trout	Canada, Qc	+	+	+	+	+	(Trudel *et al.*, 2013)
M10419-09	Brook trout	Canada, Qc	+	+	+	+	+	(Trudel *et al.*, 2013)
M9906-09	Brook trout	Canada, Qc	+	+	+	+	+	(Trudel *et al.*, 2013)
M9954-10	Brook trout	Canada, Qc	+	+	+	+	+	(Trudel *et al.*, 2013)
M8029-10	Brook trout	Canada, Qc	+	+	+	+	+	(Trudel *et al.*, 2013)
M11603-10	Brook trout	Canada, Qc	+	+	+	+	+	(Trudel *et al.*, 2013)
M6363-10	Brook trout	Canada, Qc	+	+	+	+	+	(Trudel *et al.*, 2013)
M9221-10	Brook trout	Canada, Qc	+	+	+	+	+	(Trudel *et al.*, 2013)
M10935-11	Brook trout	Canada, Qc	+	+	+	+	+	(Trudel *et al.*, 2013)
M15448-11	Brook trout	Canada, Qc	+	+	+	+	+	(Trudel *et al.*, 2013)
M16474-11	Brook trout	Canada, Qc	+	+	+	+	+	(Trudel *et al.*, 2013)
M19878-11	Brook trout	Canada, Qc	+	+	+	+	+	(Trudel *et al.*, 2013)
M11500-11	Brook trout	Canada, Qc	+	+	+	+	+	(Trudel *et al.*, 2013)
M16486-11	Brook trout	Canada, Qc	+	+	+	+	+	(Trudel *et al.*, 2013)
M13460-11	Brook trout	Canada, Qc	+	+	+	+	+	(Trudel *et al.*, 2013)
M13729-11	Brook trout	Canada, Qc	+	+	+	+	+	(Trudel *et al.*, 2013)
M14481-11	Brook trout	Canada, Qc	+	+	+	+	+	(Trudel *et al.*, 2013)
M15879-11	Brook trout	Canada, Qc	+	+	+	+	+	(Trudel *et al.*, 2013)
M17739-11	Brook trout	Canada, Qc	+	+	+	+	+	(Trudel *et al.*, 2013)
M13732-11	Brook trout	Canada, Qc	+	+	+	+	+	(Trudel *et al.*, 2013)
M17053-11	Brook trout	Canada, Qc	+	+	+	+	+	(Trudel *et al.*, 2013)
M15878-11	Rainbow trout	Canada, Qc	+	+	+	+	+	(Trudel *et al.*, 2013)
M13182-11	Atlantic salmon	Canada, Qc	+	+	+	+	+	(Trudel *et al.*, 2013)
M17735-11	Brook trout	Canada, Qc	+	+	+	+	+	(Trudel *et al.*, 2013)
M15576-11	Brook trout	Canada, Qc	+	+	+	+	+	(Trudel *et al.*, 2013)
M22710-11	Brook trout	Canada, Qc	+	+	+	+	+	(Trudel *et al.*, 2013)
M13764-11	Brook trout	Canada, Qc	+	+	+	+	+	(Trudel *et al.*, 2013)
M23911-11	Brook trout	Canada, Qc	+	+	+	+	+	(Trudel *et al.*, 2013)
M12357-12	Brook trout	Canada, Qc	+	+	+	+	+	This study
M21375-12	Brook trout	Canada, Qc	+	+	+	+	+	This study
M16237-12	Brook trout	Canada, Qc	+	+	+	+	+	This study

M12976-12	Brook trout	Canada, Qc	+	+	+	+	+	This study
M22895-12	Brook trout	Canada, Qc	+	+	+	+	+	This study
M10745-12	Brown trout	Canada, Qc	+	+	+	+	+	This study
M9754-12	Brook trout	Canada, Qc	+	+	+	+	+	This study
M17930-12	Brook trout	Canada, Qc	+	+	+	+	+	This study
M12418-12	Brook trout	Canada, Qc	+	+	+	+	+	This study
M21368-12	Brook trout	Canada, Qc	+	+	+	+	+	This study
M13050-12	Brook trout	Canada, Qc	+	+	+	+	+	This study
M24783-12	Brook trout	Canada, Qc	+	+	+	+	+	This study
M13566-12	Brook trout	Canada, Qc	+	+	+	+	+	This study
M14404-12	Atlantic salmon	Canada, Qc	+	+	+	+	+	This study
M16671-12	Brook trout	Canada, Qc	+	+	+	+	+	This study
M16042-12	Brook trout	Canada, Qc	+	+	+	+	+	This study
SHY13-162	Brook trout	Canada, Qc	+	+	+	+	+	This study
SHY13-574	Brook trout	Canada, Qc	+	+	+	+	+	This study
SHY13-1470	Brook trout	Canada, Qc	+	+	+	+	+	This study
SHY13-2188	Brook trout	Canada, Qc	+	+	+	+	+	This study
SHY13-2222	Brook trout	Canada, Qc	+	+	+	+	+	This study
SHY13-2257	Brook trout	Canada, Qc	+	+	+	+	+	This study
SHY13-2263	Brook trout	Canada, Qc	+	+	+	+	+	This study
SHY13-2317	Brook trout	Canada, Qc	+	+	+	-	+	This study
SHY13-2425	Brook trout	Canada, Qc	+	+	+	-	+	This study
SHY13-2458	Brook trout	Canada, Qc	+	+	+	+	+	This study
SHY13-2534	Atlantic salmon	Canada, Qc	+	+	+	+	+	This study
SHY13-2627	Brook trout	Canada, Qc	+	+	+	+	+	This study
SHY13-2630	Brook trout	Canada, Qc	+	+	+	+	+	This study
SHY13-2825	Brook trout	Canada, Qc	+	+	+	+	+	This study
SHY13-2873	Brook trout	Canada, Qc	+	+	+	+	+	This study
SHY13-2909	Brook trout	Canada, Qc	+	+	+	+	+	This study
SHY13-3101	Brook trout	Canada, Qc	+	+	+	+	+	This study
SHY13-3127	Brook trout	Canada, Qc	+	+	+	+	+	This study
SHY13-3795	Brook trout	Canada, Qc	+	+	+	+	-	This study
SHY13-3798	Brook trout	Canada, Qc	+	+	+	+	+	This study
SHY13-3799	Brook trout	Canada, Qc	+	+	+	+	+	This study
SHY14-2246	Brook trout	Canada, Qc	+	+	+	+	+	This study
SHY14-4161	Brook trout	Canada, Qc	+	+	+	-	+	This study
SHY14-3290	Brook trout	Canada, Qc	+	+	+	+	+	This study
SHY14-3402	Brook trout	Canada, Qc	+	+	+	+	+	This study
SHY14-2420	Brook trout	Canada, Qc	+	+	+	+	+	This study
SHY14-1503	Brook trout	Canada, Qc	+	+	+	+	+	This study
SHY14-710	Brook trout	Canada, Qc	+	+	+	+	+	This study
SHY14-2996	Arctic Char	Canada, Qc	+	+	+	+	+	This study
SHY14-2939	Brook trout	Canada, Qc	+	+	+	+	+	This study

SHY14-3674	Brook trout	Canada, Qc	+	+	+	+	+	This study
SHY14-3502	Brook trout	Canada, Qc	+	+	+	-	+	This study
SHY14-2928	Brook trout	Canada, Qc	+	+	+	+	+	This study
SHY14-2485	Brook trout	Canada, Qc	+	+	+	+	+	This study
JF2869	Arctic Char	Eur	-	+	+	-	+	This study
HER1085	Trout	Eur, Norway	+	+	+	+	+	(Trudel *et al.*, 2013)
HER1108	-	Eur, Denmark	-	+	+	+	+	(Trudel *et al.*, 2013)
HER1104	-	Eur, France	-	+	+	+	***	(Trudel *et al.*, 2013)
HER1084	-	Eur, France	-	+	+	+	-	(Trudel *et al.*, 2013)
A449	Brown trout	Eur, France	+	+	+	+	-	(Trudel *et al.*, 2013)
RS 534	-	Eur, France	-	+	+	+	-	This study
JF2506	Atlantic salmon	Eur, Norway	+	+	+	+	***	This study
JF2510	Atlantic salmon	Eur, Norway	+	+	+	+	+	This study
JF3517	Turbot	Eur, Norway	+	+	+	-	+	This study
JF3518	Turbot	Eur, Norway	+	+	+	-	+	This study
RS 887	Coho salmon	Eur, Russia	-	+	+	+	+	This study
JF2267	Arctic Char	Eur, Swit.	+	+	+	-	+	This study
JF3224	Brown trout	Eur, Swit.	-	+	+	+	+	This study
JF3327	Arctic Char	Eur, Swit.	+	+	+	+	+	This study
JF3496	Brown trout	Eur, Swit.	+	+	+	+	+	This study
JF3844	Arctic Char	Eur, Swit.	+	+	+	+	+	This study
JF3223	White Fish	Eur, Swit.	+	+	+	+	+	This study
JF3519	Arctic Char	Eur, Swit.	-	+	+	+	+	This study
JF3791	Arctic Char	Eur, Swit.	-	+	+	+	-	This study
JF4111	Arctic Char	Eur, Swit.	+	+	+	-	+	This study
JF4112	Arctic Char	Eur, Swit.	+	+	+	-	+	This study
JF4113	Arctic Char	Eur, Swit.	+	+	+	-	+	This study
JF4114	Arctic Char	Eur, Swit.	+	+	+	-	+	This study
RS 530	Atlantic salmon	Eur, UK	-	+	+	+	+	This study
JF2507	Atlantic salmon	Eur, UK	+	+	+	+	***	This study
JF3507	Atlantic salmon	Eur, UK	-	+	+	+	+	This study

a: The genome of the isolates highlighted in yellow were sequenced using Illumina technology.
b: New Brunswick (NB), Nova Scotia (NS), Québec (Qc), Ontario (On), Europe (Eur), Switzerland (Swit.), United Kingdom (UK).
c: The presence of a the TTSS region is based on the analysis of the *acrV* gene by PCR. The presence is indicated by "+" and the absence by "-" highlighted in red.
d: The presence of a plasmid is indicated by "+" and the absence by "-" highlighted in red. Newly identified plasmid variants are indicated by "***"highlighted in blue.

Trudel, M. V, Tanaka, K. H., Filion, G., Daher, R. K., Frenette, M., and Charette, S. J. (2013). Insertion sequence AS5 (ISAS5) is involved in the genomic plasticity of *Aeromonas salmonicida*. *Mob. Genet. Elements* 5, 1–7. doi:10.4161/mge.25640
