# Supplementary material for: Diversity and Homogeneity among Small Plasmids of Aeromonas salmonicida subsp. salmonicida Linked with Geographical Origin
Source: Front Microbiol. 2015 Nov 23;6:1274. doi: 10.3389/fmicb.2015.01274 (PMC4655240; doi:10.3389/fmicb.2015.01274)
Supplement: Table S2 — Primers used for the PCR genotyping. [file Table2.PDF]

Table S2. Primers used for the PCR genotyping

Genomic target	Sequence (5'-3')	Gene targeted	Reference
pAsa1	F : GGACGATTAACCTTCGCATC<br>R : GTATCGCCCAACTTCTTCCA	*relB/relE*	(Boyd *et al*., 2003)
pAsa2	F : AAAAGAGCGTGCAACCCTAA<br>R : GCGATGCTACTTCATTCACC	*relE/relB*	(Boyd *et al*., 2003)
pAsa3	F : TCATGGAGAATGTTCGCAAG<br>R : GCCCAATTATCACAGCAACA	*orf3*	(Boyd *et al*., 2003)
pAsal1	F : TAACATGGGTGAGTCAGGA<br>R :TGCATGTTTGTAAAAAGTAGGTG	*aopP*	(Boyd *et al*., 2003)
TTSS	F: GTAAAGGGTTGCGGGATGAG<br>R : GCCGCTCTTCTTCAGGTCAC	*acrV*	(Ebanks *et al*., 2006)
IS*AS11*	F: GAGCGCGTTGCAAAAGGTGTGCAG<br>R : GTTTGATACATCCAATGGGAAGGG	IS*AS11*	This study
Chromosome	F: ACATGAAGAAGCAATCAGGC<br>R : AGAGGTCATGCGTTAGCAG	*tapA*	(Ebanks *et al*., 2006)

Boyd, J., Williams, J., Curtis, B., Kozera, C., Singh, R., and Reith, M. (2003). Three small, cryptic plasmids from *Aeromonas salmonicida* subsp. *salmonicida* A449. *Plasmid* 50, 131–144. doi:10.1016/S0147-619X(03)00058-1.

Ebanks, R. O., Knickle, L. C., Goguen, M., Boyd, J. M., Pinto, D. M., Reith, M., and Ross, N. W. (2006). Expression of and secretion through the *Aeromonas salmonicida* type III secretion system. *Microbiology* 152, 1275–1286. doi:10.1099/mic.0.28485-0.
