# Supplementary material for: Diversity and Homogeneity among Small Plasmids of Aeromonas salmonicida subsp. salmonicida Linked with Geographical Origin
Source: Front Microbiol. 2015 Nov 23;6:1274. doi: 10.3389/fmicb.2015.01274 (PMC4655240; doi:10.3389/fmicb.2015.01274)
Supplement: Table S3 — Sequence differences (SNPs and InDels) found in the small plasmids of the sequenced strains of A. salmonicida. [file Table3.PDF]

Table S3. Sequence differences (SNPs and InDels) found in the small plasmids of the sequenced strains of *A. salmonicida*

Isolate	pAsa1	pAsa2	pAsa3	pAsal1
**2009-157K5**	No alteration	No alteration	No alteration	No alteration
**2010-47K18**	No alteration	No alteration	No alteration	No alteration
**2004-05MF26**	No alteration	No alteration	No alteration	No alteration
**2004-208**	No alteration	No alteration	Currently impossible to assemble pAsa3	No alteration
**RS 1752**	No alteration	Extension (43-bp duplication) (2631-2632)	No alteration	No alteration
**01-B526**	No alteration	G->A (192)	No alteration	No alteration
**08-2647**	No alteration	No alteration	No alteration	No alteration
**08-2783**	No alteration	No alteration	No alteration	No alteration
**M11743-09**	No alteration	No alteration	G->T (3056) in HP and causes a (R->S)	No alteration
**M16474-11**	No alteration	No alteration	G->T (3056) in HP and causes a (R->S)	No alteration
**M19878-11**	No alteration	No alteration	G->T (3056) in HP and causes a (R->S)	No alteration
**M17053-11**	No alteration	No alteration	No alteration	No alteration
**M22710-11**	No alteration	No alteration	G->T (3056) in HP and causes a (R->S)	No alteration
**M17930-12**	No alteration	No alteration	No alteration	No alteration
**M13566-12**	No alteration	No alteration	No alteration	Deletion of 184 bps in *mobA* (1630..1813)
**SHY13 2222**	No alteration	No alteration	G->T (3056) in HP and causes a (R->S)	No alteration
**SHY13 2317**	No alteration	No alteration	Absent	No alteration
**SHY13 2425**	No alteration	No alteration	Absent	No alteration
**SHY13 3795**	No alteration	No alteration	No alteration	Absent
**HER1085**	No alteration	No alteration	No alteration	No alteration
**RS 534**	No alteration	No alteration	C->A in *mobA* and *mobB* (5136), synonymous in *mobA* but (A->E) in *mobB*.	Absent
**JF2506**	No alteration	No alteration	Deletion of 16 bps (4793..4808) in *mobA* and *mobB*	pAsal1C
**JF3517**	No alteration	C->T (2796)	Absent	No alteration
**JF3224**	No alteration	No alteration	No alteration	No alteration
**JF3791**	C->T (3270)	No alteration	No alteration	Absent
**JF2507**	No alteration	No alteration	G->T (3435)	pAsal1D
**A449**[a]	No alteration	No alteration	G->C (2838 and 2859) in *rep,* both cause a (A->P). C->A in *mobA* and *mobB* (5136), synonymous in *mobA* but (A->E) in *mobB*	Absent
**JF2267**[b]	N/A	NA	N/A	C->G (1448) and G->A (1450), same codon in *mobA* (Q->E)

a: Analysis based on sequences published in Boyd *et al.*, 2003.
b: Analysis based on the sequence of pAsal1 published in Fehr *et al.*, 2006. The other small plasmids of this strain were not analyzed in the study by Fehr *et al.*

Fehr, D., Casanova, C., Liverman, A., Blazkova, H., Orth, K., Dobbelaere, D., Frey, J., and Burr, S. E. (2006). AopP, a type III effector protein of *Aeromonas salmonicida*, inhibits the NF-kappaB signalling pathway. *Microbiology* 152, 2809–2818. doi:10.1099/mic.0.28889-0.
