# Supplementary figures and images for: Diversity and Homogeneity among Small Plasmids of Aeromonas salmonicida subsp. salmonicida Linked with Geographical Origin
Source: Front Microbiol. 2015 Nov 23;6:1274. doi: 10.3389/fmicb.2015.01274 (PMC4655240; doi:10.3389/fmicb.2015.01274)

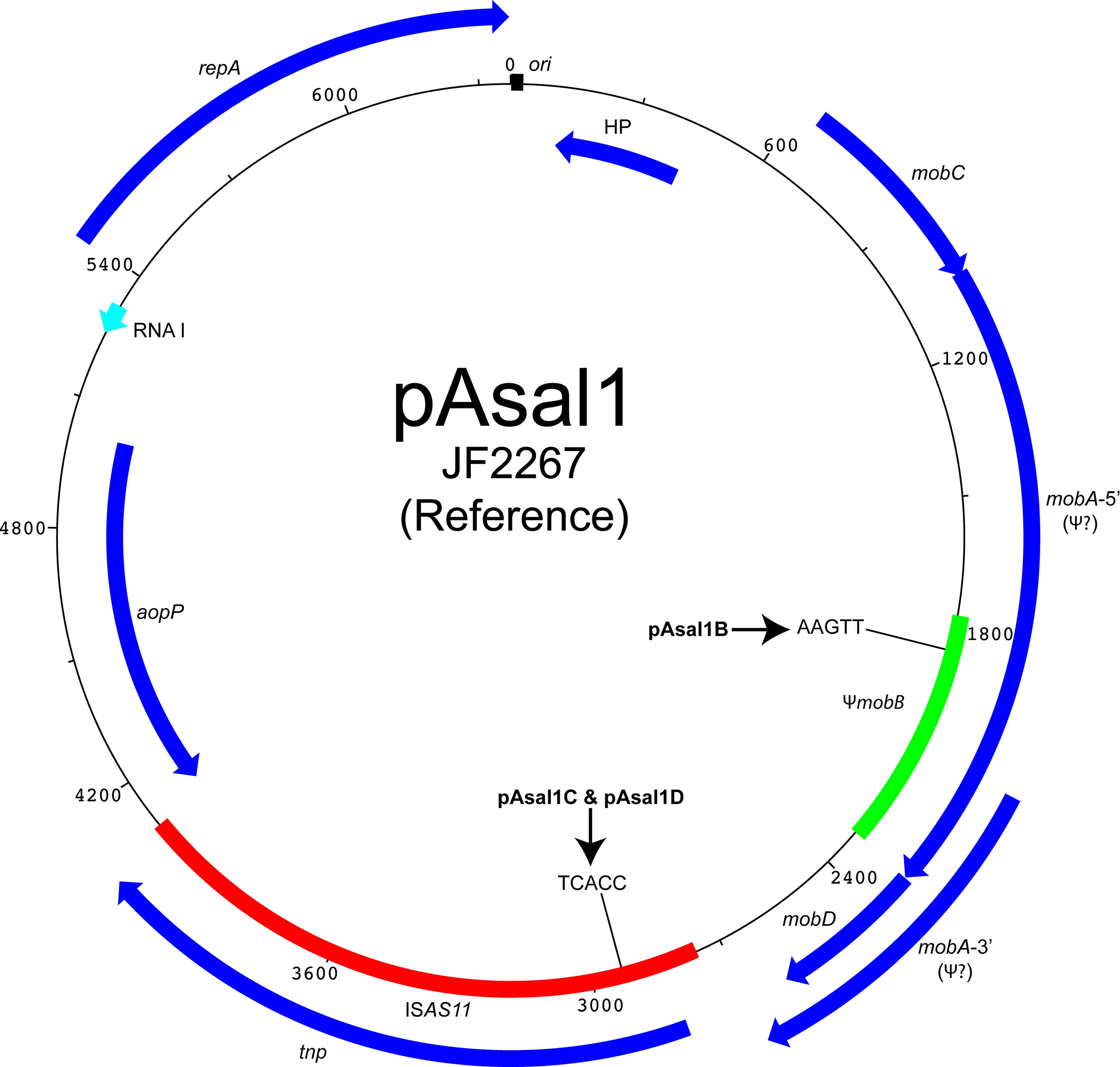

Supplement: Figure S1 — Map of the reference pAsal1 plasmid and the insertion sites of the ISAS5s in pAsal1B, pAsal1C, and pAsal1D. The arrows in dark and light blue represent the coding sequences and the RNA I regulator, respectively. The red and green rectangles represent the ISAS11 and the putative pseudogene mobB, respectively. The insertion sites (and their sequences) for the ISAS5s in all pAsal1B-C-Ds are shown. [file Image1.TIF]

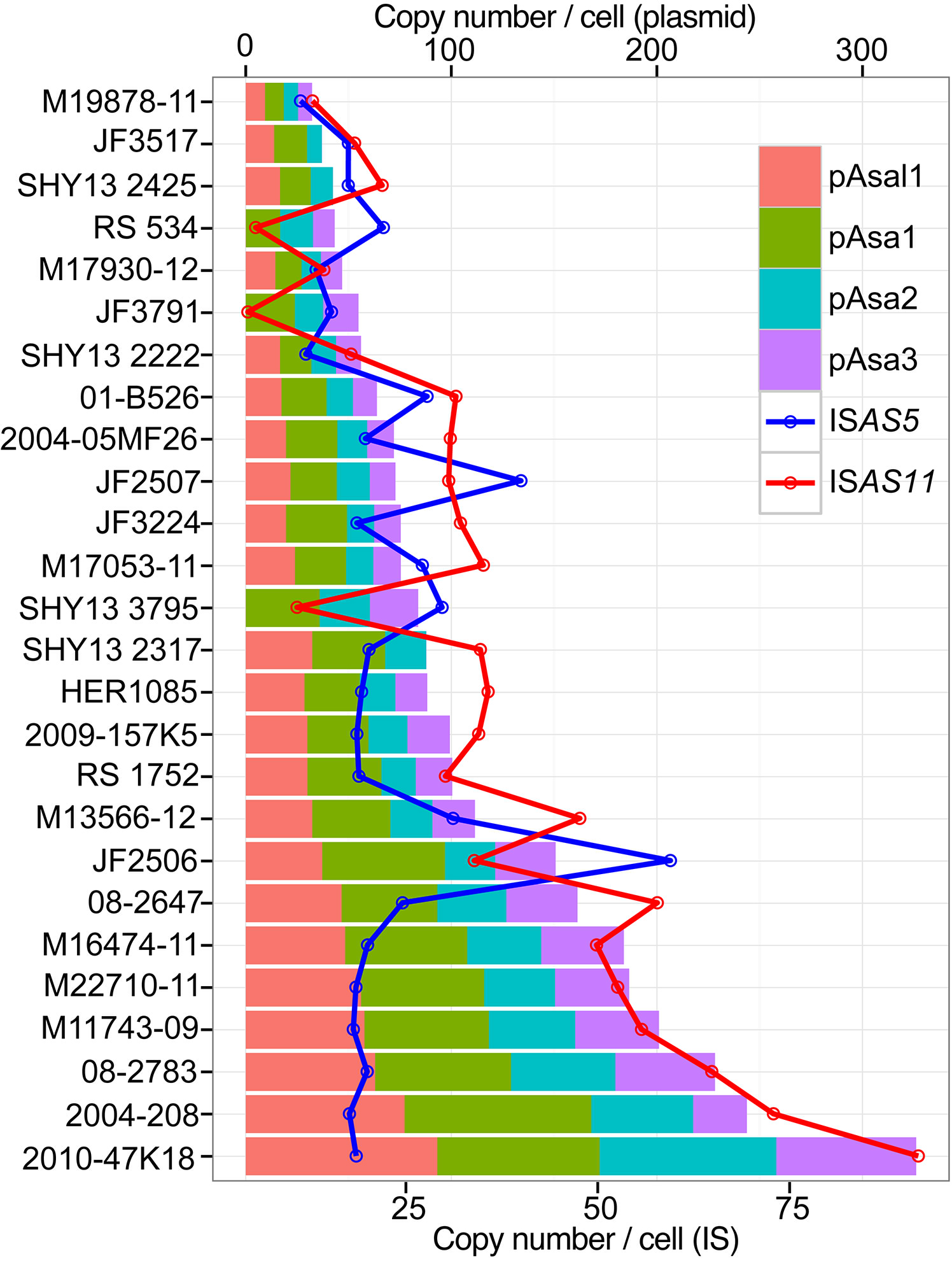

Supplement: Figure S2 — Average copy numbers of pAsal1, pAsa1, pAsa2, and pAsa3 for all the isolates for which the DNA was sequenced. The copy numbers for ISAS5 and ISAS11 were also computed since they are found on pAsal1 (and its variants for ISAS5). As indicated in the main article, the coverages provided by the sequencing reads were used to infer the information on the average copy number for each plasmid and IS. [file Image2.TIF]

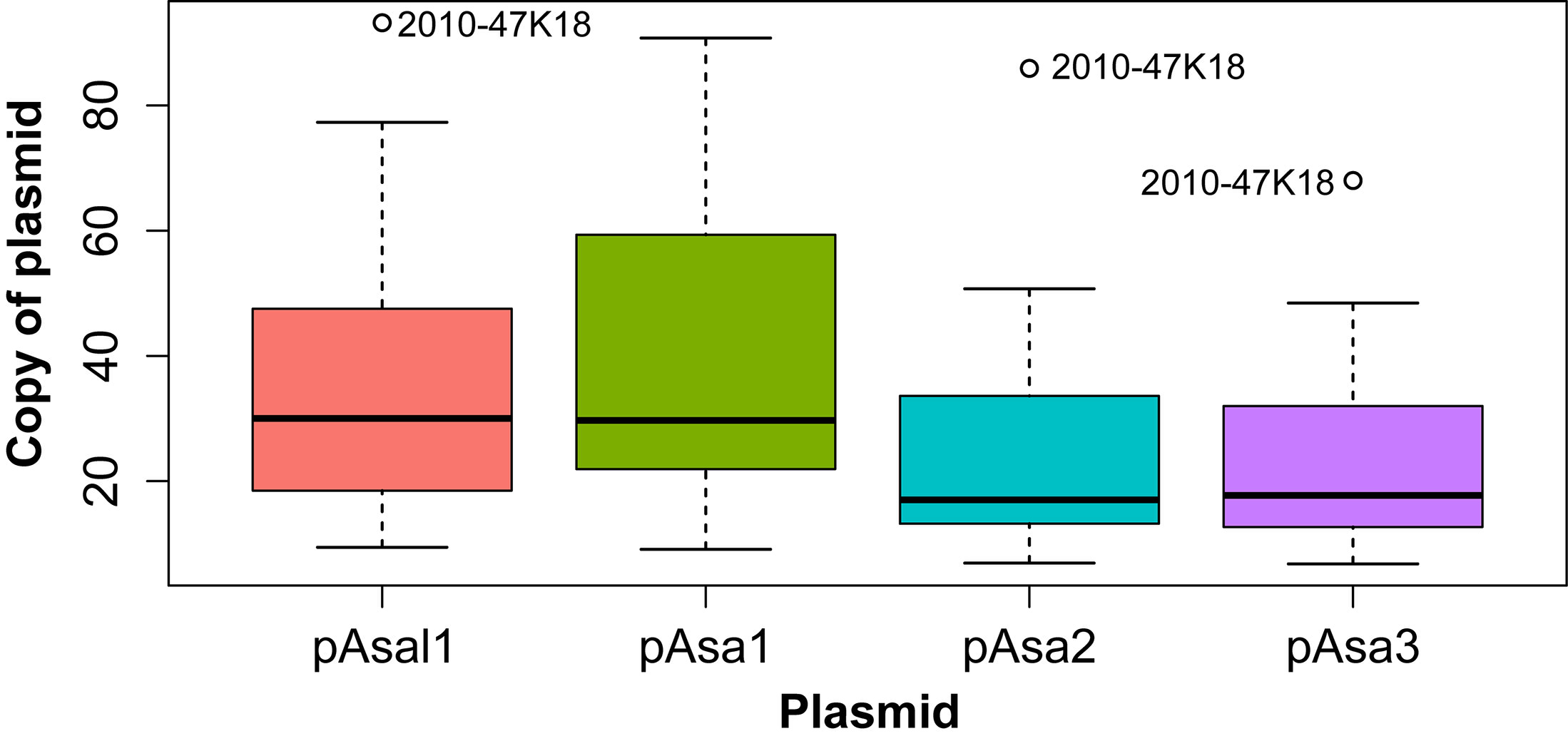

Supplement: Figure S3 — Boxplots of the average copy number of each plasmid found in the strains presented in Figure S2. [file Image3.TIF]

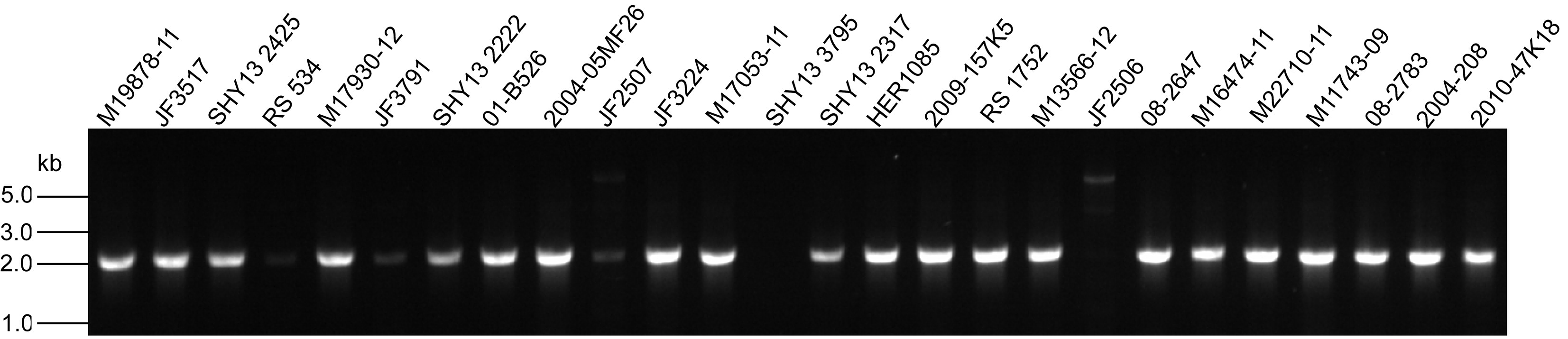

Supplement: Figure S4 — PCR amplifications of the ISAS11 in pAsal1 of all 26 strains for which the genomic DNA was sequenced. [file Image4.TIF]
